# A bidirectional reversible and multilevel location privacy protection method based on attribute encryption

**DOI:** 10.1371/journal.pone.0309990

**Published:** 2024-09-06

**Authors:** Zhaowei Hu, Kaiyi Hu, Milu Md Khaled Hasan

**Affiliations:** 1 School of Computer Science and Artificial Intelligence, Changzhou University, Changzhou, China; 2 Junior High School Affiliated to Changzhou Institute of Educational Science, Changzhou, China; National University of Sciences and Technology, UNITED KINGDOM OF GREAT BRITAIN AND NORTHERN IRELAND

## Abstract

Various methods such as k-anonymity and differential privacy have been proposed to safeguard users’ private information in the publication of location service data. However, these typically employ a rigid “all-or-nothing” privacy standard that fails to accommodate users’ more nuanced and multi-level privacy-related needs. Data is irrecoverable once anonymized, leading to a permanent reduction in location data quality, in turn significantly diminishing data utility. In the paper, a novel, bidirectional and multi-layered location privacy protection method based on attribute encryption is proposed. This method offers layered, reversible, and fine-grained privacy safeguards. A hierarchical privacy protection scheme incorporates various layers of dummy information, using an access structure tree to encrypt identifiers for these dummies. Multi-level location privacy protection is achieved after adding varying amounts of dummy information at different hierarchical levels N. This allows for precise control over the de-anonymization process, where users may adjust the granularity of anonymized data based on their own trust levels for multi-level location privacy protection. This method includes an access policy which functions via an attribute encryption-based access control system, generating decryption keys for data identifiers according to user attributes, facilitating a reversible transformation between data anonymity and de-anonymity. The complexities associated with key generation, distribution, and management are thus markedly reduced. Experimental comparisons with existing methods demonstrate that the proposed method effectively balances service quality and location privacy, providing users with multi-level and reversible privacy protection services.

## 1. Introduction

Various applications and services utilizing mobile devices have emerged alongside the continuous advancement of mobile networks. Location-based services (LBS) have gained particularly significant traction. Although LBS offer convenience, they also tend to result in users inadvertently leaving extensive location and trajectory data on service platforms. This data, if analyzed and combined with additional background knowledge by malicious third parties, can severely compromise users’ privacy. For instance, attackers might deduce a user’s health conditions by analyzing queries made near medical facilities.

Researchers have developed several methods to mitigate the privacy risks associated with LBS, including location confusion, trajectory offset, dummy information, and k-anonymity. However, these methods generally adhere to a rigid “all-or-nothing” privacy standard, offering either complete and uniform privacy protection or none at all. This fails to meet users’ demands for personalized and multi-level privacy protection. Data cannot be restored to its original state after it has been anonymized, rendering it less useful for a variety of user requirements.

Existing privacy protection methods are predominantly single-layered and coarse-grained, providing a uniform level of privacy that fails to support personalized or multi-level protection. Moreover, these methods are typically unidirectional and irreversible. Dummy data cannot be removed once it is integrated into the dataset, leading to a permanent reduction in data quality and decreased utilization efficiency. While some methods do offer reversible privacy protection, their excessively complex data encryption processes and anonymity algorithms can significantly impede data processing.

To address these issues, this paper proposes a bidirectional, multi-layer reversible location privacy protection method based on attribute encryption. This method provides layered, bidirectional, and fine-grained privacy safeguards. Multi-level privacy protection for location data is achieved through a hierarchical privacy scheme that incorporates varying levels of dummy information. It utilizes ciphertexts of dummy information identifiers to control the degree of de-anonymization based on users’ individual trust levels, enabling reversible transformations between data anonymization and de-anonymization. Furthermore, the method uses an attribute-based encryption access control system to manage resources and streamline key generation and distribution, further enhancing the granularity of privacy protection.

The proposed method is applicable three distinct scenarios: when users with varying trust levels access the same data resources, when user identity must remain unknown for granting permissions, or when anonymized data needs to be restored. Thus, users with different trust levels can obtain data with varying degrees of precision from the same anonymized dataset. Even without prior knowledge of a user’s identity, access control authorization is necessary. Importantly, this ensures that data anonymization does not lead to a permanent degradation of data quality, allowing for the restoration of anonymous data to its original state.

The main contributions of this work can be summarized as follows:

Firstly, a multi-level location privacy protection method is proposed that addresses the limitations of “all-or-nothing” privacy standards. It includes multiple privacy levels, incorporating varying levels of dummy information and generating a series of dummy information identifiers for multi-level protection of private location data. This method enhances privacy protection effectiveness by constructing a position adjacency table and selecting random location points via a hash function. Dummy information identifiers are encrypted using an access structure tree, which controls the degree of de-anonymization based on users’ trust levels, thus balancing privacy protection with data utilization efficiency.

Secondly, a novel bidirectional method is introduced that resolves the issue of irreversible data loss. An access policy is defined using an attribute encryption access control mechanism, incorporating an access structure tree, where user attributes are employed as encryption parameters. A trusted third party authenticates user attributes and generates decryption keys for the ciphertext of the identifier files, enabling privileged users to perform de-anonymization operations. This allows for reversible transformations between data anonymization and de-anonymization, streamlines resource control, and reduces complexities associated with key generation and distribution, thus achieving fine-grained privacy protection.

Thirdly, experiments conducted on real datasets confirm the feasibility and effectiveness of the proposed method. By comparison against existing methods, it is shown to offer more efficiently safeguard user location and trajectory data while ensuring bidirectional, multi-level, and multi-granular privacy protection.

The rest of paper is as follows: section 2 introduces the related work, section 3 systematically introduces the research contents and methods, and the safety is analyzed in section 4. The experiment and result analysis are shown in section 5. The section 6 summarizes the research contents of the paper and puts forward the next research direction.

## 2. Related work

LBS provide remarkable convenience in users’ daily lives but also pose significant risks due to the potential leakage of private locations and movement trajectories. Researchers have developed various privacy protection methods to address these concerns, such as confusion techniques, location offsets, and dummy information. Among these, the k-anonymity method is known to effectively balance data availability and privacy security, while the differential privacy protection method is noted for its strict data model; both have become important topics of research in this field.

Other methods, like query semantic analysis, have been found to undermine anonymity. For instance, Yang et al. [1] proposed a dual privacy protection scheme based on a multi-anonymous architecture. This method encrypts queries via the Shamir mechanism and enhances privacy by replacing sensitive semantic locations with anonymous sets that reflect user diversity. However, the encryption and decryption of query content can create excessive response times and diminish quality of service.

Wang et al. [2] proposed an L-clustering algorithm based on differential privacy protection, which clusters users’ locations based on duration of stay, frequency, and sensitivity while incorporating Laplacian noise for privacy protection. However, this method’s consumption of privacy budget parameters is burdensome. Xing et al. [3] developed a distributed k-anonymous location scheme that forms anonymized groups based on users’ interests and social behaviors, reducing the risk of attacks leveraging background knowledge. These methods often rely on a central trusted server for data anonymization, however, which raises concerns about potential data breaches. This underscores the demand for more innovative, distributed privacy protection approaches.

The decentralization aspect of blockchain technology offers novel solutions for privacy protection. Zhang et al. [4] suggested a method based on the (t, n) threshold scheme and smart contracts, encrypting and distributing user queries via a private blockchain and the Shamir algorithm to prevent collusion attacks. This method also incentivizes timely submission of anonymous queries through smart contracts. Although this approach integrates blockchain technology, it falls short of achieving a fully decentralized LBS. In addressing the risk of privacy breaches by untrusted collaborators and the leakage of semantic location information, Yang et al. [5] introduced a mechanism that combines blockchain with a user-related semantic location model. It leverages public chains for issuing privacy requests and private chains for selecting anonymous locations, using smart contracts to enhance the security of the collected semantic information. However, this method lack clarity in the implementation of private chains and smart contracts, which may hinder its practical application.

Additionally, Zhu et al. [6] proposed a blockchain-based scheme for privacy-preserving location-sharing, in which precise locations are converted into broader areas; sharing details are varied based on the trust level of the requester with a Merkle tree for data segmentation. Shen et al. [7] proposed combining blockchain and machine learning technologies to securely store transaction and trust data, thereby protecting against malicious tampering and addressing other significant privacy concerns associated with the Internet of Vehicles.

Despite their utility in some regards, the prevailing privacy protection methods generally adhere to a rigid “all-or-nothing” standard whereby they either provide complete and uniform privacy protection or none at all. This fails to address users’ needs for personalized and multi-level privacy options. Moreover, these systems do not allow user location data to be reverted to its original form once it has been anonymized, leading to irreversible loss of data quality and negatively impacting data utilization efficiency.

Li et al. [8–10] proposed a reversible location anonymity method designed to restore location data for mobile device users. Their method employs a spatiotemporal anonymity model to reversibly alter location data, achieving high spatial resolution and commendable success rates. However, the complexity of the data encryption process and the choice of anonymity algorithms compromise data processing efficiency. The method continuously reconstructs links from previously selected ones during the anonymization process, adjusting selections based on current conditions and thus, unfortunately, creating excessive temporal complexity. On the one hand, it requires lengthy anonymization runtimes, while constructing conflict-free links in real-time. And on the other, it demands significant memory space foe storing conflict-free links, similarly failing to meet users’ requirements for real-time and efficient privacy protection.

Buccafurri et al. [11] introduced a hierarchical location-based trusted service scheme based on the edge cloud paradigm, which distributes user information among hierarchical regions managed by different autonomous organizations. Lower-level services manage exact location data, whereas higher-level services manage only aggregated data, which addresses the potential privacy leaks caused by centralized service failures.

Though these methods secure user location data in LBS, they do not offer multi-granularity privacy protection tailored to actual user needs nor do they support reversible or fine-grained safeguarding. Moreover, after anonymizing the data, it cannot be restored to the original state, which will seriously affect the efficiency of data. Other relevant privacy protection methods are summarized in Table 1.

**Table 1:** Summary of relevant privacy protection methods.

Ref.	Year	Approach	Advantage	Disadvantage
[2]	2023	Differential Privacy	It can allocate privacy budget according to user’s privacy preference and generate false location range acceptable to users.	Connecting the cluster residence points and centroids may leak the user’s trajectory privacy.
[12]	2021	Random sampling	It can reduce the time complexity of data processing by randomly sampling the original data set.	It is difficult to set the right privacy budget parameters to ensure that the amount of added noise meets privacy protection and availability requirements.
[13]	2023	Markov model	It can improve hit rate and reduce interaction times by caching query data locally.	Once the cache data is attacked, it may reveal user privacy.
[14]	2023	Location Semantics	A semantic quantization representation mechanism is established to protect user’s location privacy by protecting location semantics.	The process of constructing similar trajectory vector, optimizing trajectory and adding noise is complex, which makes the time complexity is higher.
[15]	2021	K−anonymity scheme	It protects user location privacy by encrypting location information.	K-anonymity location privacy protection scheme cannot resist attacks based on background knowledge.
[16]	2022	Trajectory similarity	In the process of collecting and publishing location data, privacy protection method is introduced to realize double privacy protection.	It is difficult to set the similarity and difference between the original trajectory and the replacement trajectory, which affects the privacy protection effect.
[17]	2022	Deep learning model	It combines deep learning methods with differential privacy protection models to improve privacy protection.	Noise is added by predicted privacy budgets, there are usage errors, and it cannot be applied to static data scenarios.
[18]	2022	Dummy location	It takes into account semantic sensitivity, offset location, query probability and location distribution to achieve location privacy desensitization.	The processing process is complex and the time complexity is higher.
[19]	2022	Semantics prediction	It uses Markov model to predict attack probability to adjust privacy budget, so that privacy protection effect is better.	It is difficult to classify privacy levels, which affect privacy budgets and privacy protection.
[20]	2022	Sensitive area replacement	It protects trajectory privacy by protecting sensitive dwell areas and can reduce the number of position trajectories processed.	It adds noise based on similarities between trajectories and fails to prevent similarity attacks.
[21]	2021	Trajectory graph model	It introduces time series into location data and upgrades location data mining to frequent trajectory graph mining.	The mining process is high computational complexity and slow response.
[22]	2020	Bidirectional k-anonymity	It protects the security of user location and query location, and it performs bidirectional k-anonymity according to semantics.	The influence of similarity and difference on privacy protection effect is not considered in the process of finding the best cooperation segment.
[23]	2016	Multi-level protection	It considers query probability and data timeliness when generating anonymous regions to enhance privacy security.	Cache-ware caches may reveal users’ private information.

As discussed above, the irreversible data anonymization process severely impairs data efficiency. The current research primarily centers on anonymization, neglecting the potential for data de-anonymization, though it is crucial in practical analysis applications where de-anonymization is crucial to fully harness the value of such data. To address these shortcomings, this paper proposes a bidirectional, multi-layer reversible location privacy protection method based on attribute encryption. This method not only supports bidirectional operations but also offers multi-layered and fine-grained, personalized privacy safeguards, catering to diverse user demands and facilitating data reversibility in multi-user and multi-demand scenarios.

Important distinctions between the proposed method and existing methods are twofold: firstly, it facilitates bidirectional, reversible processing of private location data by incorporating dummy information at varying strengths across different levels. This not only provides anonymized privacy protection but also allows for the refinement of de-anonymized data. It establishes multi-level privacy protection tailored to users’ needs, enabling those with different permissions to access data at different levels of anonymity and precision. Secondly, the proposed method enhances resource control through an attribute encryption access control system. This system manages the encryption of de-anonymized identifier files and the generation and distribution of attribute keys, achieving reversible and robust privacy protection effects.

## 3. Research methodology

To achieve bidirectional, reversible, and multi-level privacy protection for mobile users’ location and trajectory data, the proposed method integrates a variety of techniques including privacy protection, data encryption, access control, and attribute encryption.

The data owner first establishes privacy protection levels and incrementally adds dummy information, generating corresponding identifier files for each level. These files catalog all dummy information incorporated at that particular level. The data owner crafts an access policy for each identifier, creates an attribute access structure tree, and uses this tree as a parameter to encrypt the identifier files, producing identifier-file ciphertexts. The data owner then transmits the final anonymized dataset along with these ciphertexts to the data service center and sends the access structure tree to a trusted third party. A privileged user, whose attributes satisfy the access criteria attached to the access structure tree, can request a decryption key from the trusted third party. Upon obtaining this key, the user is able to decrypt the relevant dummy information identifier file, carry out the de-anonymization process, and access a more precise dataset at the desired level of privacy protection.

### 3.1 Workflow

**Fig 1:**
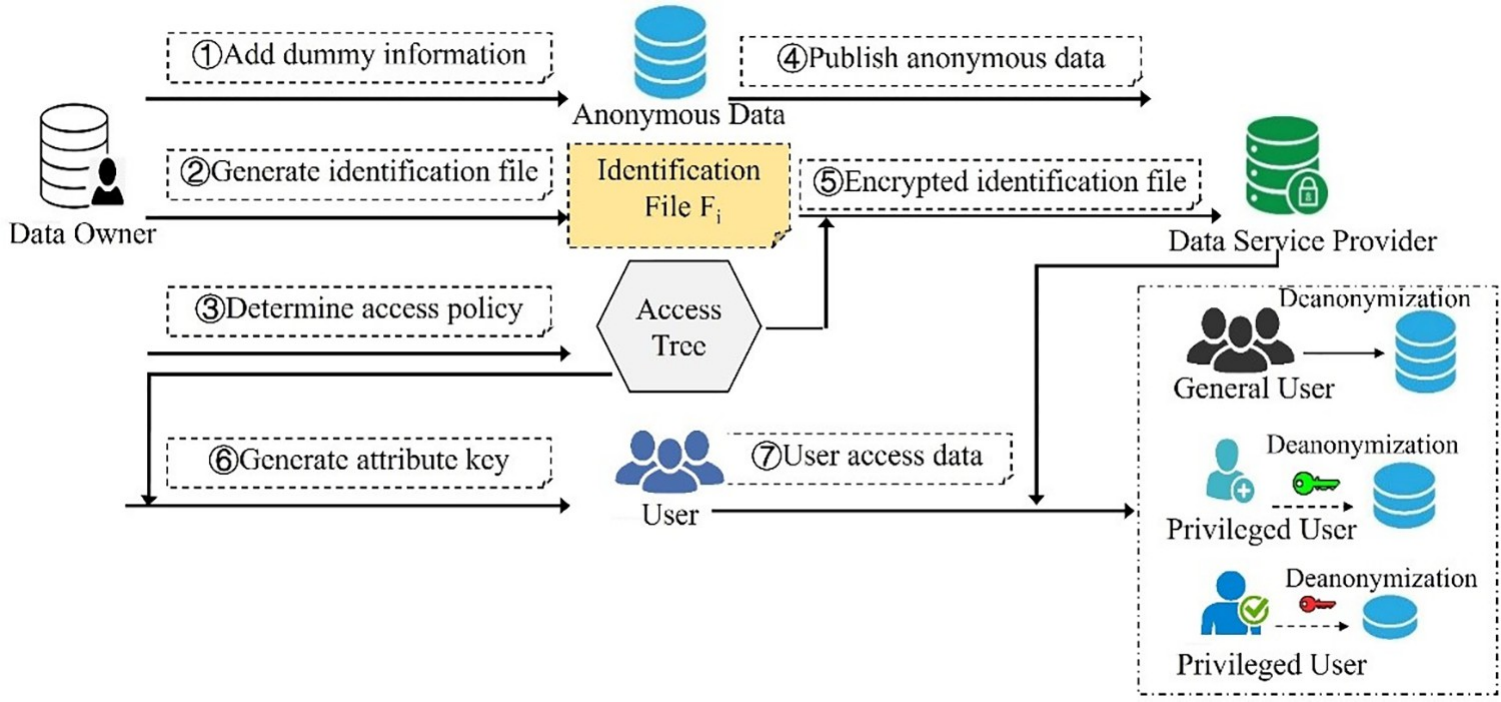
Workflow of bidirectional multi-layer anonymity processing model.

In the paper, we propose a method to achieve hierarchical privacy protection by adding dummy information layer by layer, generate and manage keys by using access control technology based on attribute encryption, and de-anonymize anonymous data sets by identifying files with dummy information, which can achieve bidirectional, reversible, multilevel and fine-grained protection of user location privacy data. The whole process includes the following seven steps: adding dummy information, generating dummy information identification files, setting access control policies, publishing anonymous data sets, encrypting identification files, generating attribute keys, and accessing data by users. The specific workflow is shown in Fig 1.

1. Adding dummy information. According to privacy protection requirements, the anonymity data is divided into N different levels, it is represented as L_0_,L_1_,L_2_,L_3_,…,L_N-1_, which satisfies the condition L_0_<L_1_<L_2_<L_3_<…<L_N-1_, where L_0_ only contains real location information, L_N-1_ is the highest degree of anonymity, i.e., the final published anonymous data set. Greedy algorithm is used to add dummy location information, and randomly adds adjacent or connected locations to the current anonymous set.
2. Generating identification files. According to the set anonymity level, while adding corresponding amount of dummy information hierarchically, the dummy information identification file is established hierarchically. The identification file B_i_ and the anonymity level L_i_ are one-to-one corresponding, and the lower anonymity level is, the more dummy location information is marked in the identification file. Then the more dummy information can be removed by de-anonymization, the more accurate data will be. The relationship between identification files is: B_1_⊇B_2_⊇B_3_⊇…⊇B_N-1_, and the number of dummy data in each level is |B_i_|. At each anonymity level, only dummy information is added and identification files are generated, and anonymous data is not published. Finally, a unified anonymous data set is generated, and N-1 identification files are generated.
3. Setting access control policies. The data owner formulates the access policy of the dummy identification file and establishes the access structure tree. Only the user who meets the attribute set can obtain the decryption key and decrypt the ciphertext of identification file. The data owner may not know the identity of the potential authorized user and the associated key set in advance, and it can even keep the anonymity of authorized users in certain scenarios.
4. Publishing anonymous data set. When the de-anonymization key is generated, it only encrypts the corresponding identification file, but does not encrypt the anonymous data set, so as to improve the efficiency of data processing. Data owner generates a unified anonymous dataset and publishes it along with a series of identifying file ciphertexts. All data users and attackers face the same anonymous data set, and privileged users can remove the corresponding level of dummy information by de-anonymizing the corresponding key.
5. Encrypting identification files. Based on the access control structure tree, the data owner encrypts the identification file to generate a series of identification file ciphertext. Only user who meets the access attribute conditions can obtain the decryption key and decrypt the identification file ciphertext at the level.
6. Generating attribute keys. According to the access control policy and the attribute certificate of the applicant user, the trusted third party generates key for decrypting ciphertext of identification files at different levels, and then sends the key to the corresponding user.
7. User access data.

For an ordinary user, because there is no key to decrypt the identification file ciphertext, he can only use unified data with high anonymity, so as to protect the privacy of users. For a privileged user, he can obtain the decryption key of the identification file ciphertext from the trusted third party, obtain relatively accurate location data to improve the efficiency of data.

### 3.2 Constructing undirected graph and adjacency table

Before the anonymization process, all road segments in the map are pre-assigned their connections in a conflict-free manner, and then it selects dummy information according to the pre-assigned connections, that is, all the road segments in the map will be pre-processed in a conflict-free manner to establish a road segment undirected graph. Then, based on the undirected graph, a corresponding adjacency table is generated, the header of the adjacency table is the connection point of the road segment, and the nodes in the adjacency table are all other connection points directly connected to the connection point.

The adjacency table consists of vertex nodes and table nodes, the structure of vertex node is D = (ID, Name, FirstName, Note), where ID represents the segment number, Name represents the segment name, FirstName represents the name of first node directly connected to the vertex, and Note is comment information which is used to record whether the node is added to the anonymous collection.

The structure of table node is E = (AdjuvexID, Info, NextName, Notes), where AdjuvexID represents the ID of node directly connected to the vertex, Info represents the relevant information field which is used to store information such as weight, and NextName is the segment pointing to the next directly connected node.

When a dummy location point is added, a pseudo-random number is generated by a random function, and then a location point is selected by a hash function:

$$H_{i}(Key)=Key_{i}\;mod|A|$$ (1)

where Key_i_ is the pseudo-random number generated, |A| is the number of nodes in the adjacency table, H_i_(Key) is used to select dummy node in the adjacency list.

When it selectes the corresponding connection point in the adjacency list, it first checks the identification bit of the node, if the identification bit is 0, the node is selected. If the identifier bit is 1, it means that the node has been added to the anonymous set, and there is a conflict between the selected nodes, then the open addressing method is used to resolve the conflict and reselect a new node, that is,

$$H_{i}(Key)=(H(Key)+d_{i})\;mod\;|A|$$ (2)

where i = 1,2,…,s, H(Key) is a hash function, d_i_ is an incremental sequence, and the value selection method adopts a linear detection hashing method, d_i_ = c×i, c = 1.

It reselects nodes by resolving conflicts, if the conflict is not resolved, it continues to select a new node by using the conflict hash function until a node-free location is selected.

**Fig 2:**
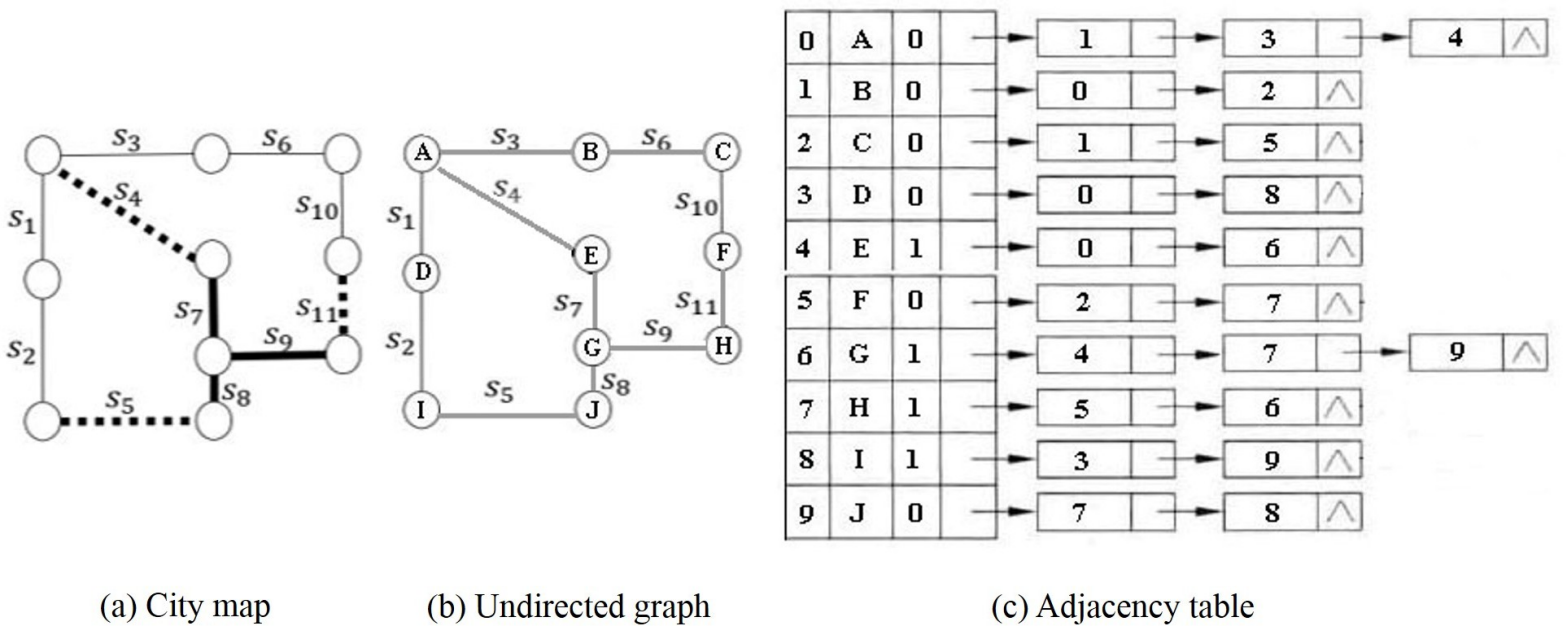
Undirected graphs and adjacency table.

After selecting the location node, it checks whether the selected connection point is directly connected to any node in the current anonymous set through the adjacency table, that is, it verifies whether the selected node is on the same adjacency table as any node in the anonymous set. If it is directly connected, the location point is added to the anonymous set, and its corresponding identification bit is marked as 1. Otherwise, there is a conflict, and it must generate new pseudo-random numbers, and then select and check the connectivity of new nodes. For example, in Fig 2A represents a city map, Fig 2B represents the constructed undirected graph, and the constructed position adjacency table is shown in Fig 2C. Therefore, for a new map, it first generates a location adjacency table corresponding to an undirected graph with algorithm 1, and then anonymizes it.

Algorithm 1: Building a Location Adjacency Graph

Input: Undirected Graph of links G = (N, E)

Output: Position Adjacency List PL

1 int i,n, e;

2 int u,v;

3 ENode **a,*t;

4 cin>>n>>e;

5 a = new ENode*[n];

6 for (i = 0;i<n;i++)

7 a[i] = NULL;

8 for (i = 0;i<e;i++) {

9 cin>>u>>v;

10 t = new ENode;

11 t->adjVex = v;

12 t->nextArc = a[u];

13 a[u] = t;

14 t = new ENode;

15 t->adjVex = u;

16 t->nextArc = a[v];

17 a[v] = t;}

18 for (i = 0;i<n;i++) {

19 cout<<“a[“<<i<<”]”;

20 t = a[i];

21 while (t! = NULL) {

22 cout<<“->”<<t->adjVex;

23 t = t->nextArc;}

24 end;}

25 return PL

### 3.3 Building anonymous datasets with multiple levels

Based on the location adjacency table, to construct anonymous data sets. During anonymous processing, each anonymous request contains a profile identifying the user’s privacy protection requirements, which contains relevant parameters for privacy protection, denoted as (L,*k*,*t*,*d*), where L represents the number of levels of anonymity protection, i.e. anonymity processing is divided into several protection levels. *k* represents the anonymity parameter, which specifies the number of other users contained in the current level of anonymity. *t* denotes a time threshold that specifies the maximum tolerated time for anonymous processing, *d* denotes the spatial threshold which specifies the range of maximum acceptable anonymous space.

In the multilevel location privacy protection model, according to the privacy protection requirements and anonymity level, the anonymity level is divided into N levels, specifically expressed as L_0_,L_1_,L_2_,L_3_,…,L_N-1_, and the anonymity parameter corresponding to the anonymity level L_i_ is:

$$k_{i}=k_{1}+(i‐1)k_{1}=i•k_{1}$$ (3)

Where 1≤i≤N, L_0_ only contains real location information, and *k*_1_ is the anonymous parameter corresponding to L_1_. The anonymity degree satisfies L_0_<L_1_<L_2_<L_3_<…<L_N-1_, L_N-1_ is the most anonymous dataset, i.e. the final published anonymous dataset.

Anonymity processing starts at L_1_, and then it constructs anonymous data sets based on anonymity-level configuration parameters (*k*_1_,*t*_1_,*d*_1_). Dummy location information is added to the data set which contains only the real user set L_0_. Firstly, *k*_1_ location points which are selected directly connected with position locations in L_0_ in the current adjacency table to construct anonymous data set M_1_ at L_1_ level. When dummy locations are added, a greedy algorithm is adopted to generate random numbers in a certain range by random function and hash function, and dummy locations are selected in the adjacency table by random numbers until the privacy configuration parameters are satisfied. If the anonymity condition of the user is still not satisfied with the set spatial threshold *d*, the anonymity is failed. Then, on the basis of L_1_, dummy information is continuously added to the anonymous set of M_1_ until the configuration parameters (*k*_2_,*t*_2_,*d*_2_) of the anonymity level of L_2_ are satisfied. At this time, the anonymous set constructed is M_2_, where M_1_⊑M_2_. It is repeated until an L_N-1_ anonymous dataset M_N-1_ is constructed, where M_1_⊑M_2_⊑M_3_⊑…⊑M_N-1_. Finally, the anonymous dataset M_N-1_ is published. The anonymous set construction process is shown in Algorithm 2.

Algorithm 2: Building an anonymous set

Input: Privacy Configuration Parameters (*k*,*t*,*d*), Anonymity Level L_i_, Adjacency List PL

Output: Anonymous set M, Identify File Set B

1 ∃ L_i_, i∈(0,N-1)

2 if i = 0

3 M_i_ = {s_0_} // s_0_ is the location of the real user

4 B_i_ = ø

5 else

6 for (i = 1; i++; i<N) {

7 k_i_ = i•k

8 H_j_(Key) = Key _j_ mod |PL| // Generate random numbers to select location points

9 ∃ s_j_∈PL, ∀s_n_∈M_i_

10 if s_j_ and s_n_ are directly connected, then M_i_ = M_i_∪ {s_j_}

11 B_i_ = B_i_∪ {s_j_}

12 count = |M_i_|

13 if count<k_i_

14 H_t_(Key) = Key _t_ mod |PL|

15 if s_t_ in PL and s_n_ in M_i_ are directly connected then M_i_ = M_i_∪ {s_t_}

16 B_i_ = B_i_∪ {s_j_}

17 count = |M_i_|

18 end if

19 B = B∪{B_i_}

20} end for

21 return M_N-1_, B

Greedy algorithm is used to add dummy location information, which randomly adds adjacent or connected locations to the current anonymous set, and each dummy location is added which connects with at least one location in the current anonymous area. In the paper, the proposed method can effectively prevent the location information without connection from being added by hash function and adjacency table, to improve the effect of privacy protection. Because it uses a random function to select locations, all locations are selected with the same probability, so an attacker or data consumer cannot determine the exact location of the real user.

***Example 1***. In Fig 2 of the above example, assuming that the road segment s_7_ contains the real location of the user, the set initially formed that only contains the real location is M_0_ = {s_7_}, and it can be used as the anonymity level L_0_. If the initial privacy protection parameter is *k* = 3 and *k* is incremented for each level thereafter, the anonymity parameter of anonymity level L_i_ is *k*_i_ = *k*+(i-1)*k* = i•k, i.e. *k*_i_ = 3+(i-1)•3 = 3i.

For anonymity level L_1_, *k*_1_ = i•*k* = 1•3 = 3, it selects a location point H in the location adjacency table which is directly connected to location point G in s_7_ by a random function and a hash function, and the identification bit of the corresponding location point is set to 1. Then the corresponding segment s_8_ is added to the anonymous set, and the anonymous set is M_1_ = {s_7_, s_8_}. At this time |M_1_|<*k*_1_, it continues to add dummy location information. It selects the location point J which is directly connected to the location point G or H in s_7_ in the location adjacency table, and the identification bit of the corresponding location point is set to 1. Then the corresponding segment s_9_ is added to the anonymous set M_1_ = {s_7_, s_8_, s_9_}, at this time |M_1_| = k_1_, the anonymity processing is completed, and the corresponding anonymity level is L_1_.

For anonymity level L_2_, k_2_ = i•k = 2•3 = 6, it selects location point A which is directly connected with location point in {s_7_, s_8_, s_9_} in location adjacency table, and the identification bit of its corresponding location point is set to 1. The corresponding segment s_4_ is added to the anonymous set, the anonymous set is M_2_ = {s_7_, s_8_, s_9_, s_4_}, where |M_2_|<k_2_, it continues to add dummy location information. Then, through random function and hash function, it selects position points F and I which are directly connected with location points in {s_7_, s_8_, s_9_, s_4_}, and it sets the identification bit of the corresponding location point to 1. The corresponding segments s_5_ and s_11_ are added to the anonymous set, the anonymous set is M_2_ = {s_7_, s_8_, s_9_, s_4_, s_5_, s_11_}, where |M_2_| = k_2_, the anonymity processing of this anonymity level is completed, and the corresponding anonymity level is L_2_.

For anonymity level L_3_, k_3_ = i•k = 3•3 = 9, it uses the same method to construct the anonymous set M_3_ = {s_7_, s_8_, s_9_, s_4_, s_5_, s_11_, s_2_, s_3_, s_10_}. When |M_3_| = k_3_, the anonymity processing is completed, and the corresponding anonymity level is L_3_.

Finally, four levels of anonymous sets are formed. For L_0_, only the real user location is contained. From L_1_ to L_3_, the degree of anonymity is gradually enhanced, and more and more dummy information is added to the anonymous set. Specifically expressed as:

L_0_: M_0_ = {s_7_}

L_1_: M_1_ = {s_7_, s_8_, s_9_}

L_2_: M_2_ = {s_7_, s_8_, s_9_, s_4_, s_5_, s_11_}

L_3_: M_3_ = {s_7_, s_8_, s_9_, s_4_, s_5_, s_11_, s_2_, s_3_, s_10_}

3.4 The de-anonymized dummy information identifiers file is created

The dummy information identification file is mainly used to identify the added dummy information at each anonymity level hierarchically, so that the corresponding dummy information can be accurately removed in the de-anonymization stage to obtain accurate data with different precisions. Therefore, it can ensure the reversibility of anonymous data and the bidirectional nature of anonymity process. The identification file corresponds to the anonymity level, i.e. each anonymity level corresponds to an identification file.

The structure of the identification file is B = (ID,L,FID), where ID represents the serial number, L represents the degree of anonymity, and FID represents the identification of added dummy information. According to the anonymity level L_1_<L_2_<L_3_<…<L_N-1_, while adding corresponding amount of dummy information hierarchically, the identification file of the dummy information is hierarchically established, and the identification file B_i_ is corresponded to the anonymity level L_i_ one by one. The lower anonymity level, the more dummy location information is marked in the identification file. The more dummy information is removed by deanonymization, the more accurate the data is obtained.

The relationship between identification files is B_1_⊇B_2_⊇B_3_⊇…⊇B_N-1_, and the number of dummy data in each level is |B_i_|. Each anonymity only adds dummy information and generates identification files, without publishing anonymous data. Finally, a unified anonymous data set is generated, and N-1 identification files are generated.

The identification file only marks the hierarchical level of privacy protection and the ID number of added dummy information. In order to improve the efficiency of encryption and decryption, only the identification file is encrypted. Then it is sent to the data server without encrypting the data, which is anonymized and distributed directly to all users.

***Example 2***. In the above example, an anonymous set with four anonymity degrees L_0_, L_1_, L_2_, and L_3_ is constructed, and the final anonymous set is M = {s_7_, s_8_, s_9_, s_4_, s_5_, s_11_, s_2_, s_3_, s_10_}. Where L_0_ only contains real users, and the identification file sets corresponding to the other three levels are: B_1_ = {s_8_, s_9_, s_4_, s_5_, s_11_, s_2_, s_3_, s_10_}、B_2_ = {s_4_, s_5_, s_11_, s_2_, s_3_, s_10_}、B_3_ = {s_2_, s_3_, s_10_}. Each identification file marks the added dummy location information to the final anonymous collection. The higher anonymity of the identification file, the less dummy location information is marked. For example, L_3_ is more anonymous than L_2_, then the dummy information labeled is added in B_3_ which is in the basis of L_2_, and the number of dummy information labeled is significantly less than B_2_.

### 3.5 Set an access strategy

In the paper, we propose a method to encrypt data by using attribute set as encryption parameters, and only user who meets the attribute set can obtain private key and decrypt data.

***Definition* 1** Attribute. It assumes that $A=\{A_{1},A_{2},\ldots ,A_{n}\}$ is the set of all attributes, then each attribute S is a non-empty subset of A, $S\in \{A_{1},A_{2},\ldots ,A_{N}\}$, then *n* attributes can identify 2^n^ users.

***Definition 2*** Access Structure. It assumes that {P_1_, P_2_,…, P_n_} is a set of participants, let $S\subseteq 2^{\left\{P_{1},P_{2},\ldots ,P_{n}\right\}}$, if ∀ B,C, then B∈S, and *B*⊆*C*, then C∈S, S is said to be monotonic. S is called an access structure if S is monotonic and nonempty, $S\subseteq 2^{\left\{P_{1},P_{2},\ldots ,P_{n}\right)}\{\emptyset \}$, and the elements of S are called authorized sets.

Access structure is mainly divided into threshold structure, attribute value and operation structure, access tree structure and LSSS matrix structure. At present, access tree structure is widely used in access control, which can be regarded as an extension of single-layer (t,n) threshold structure and supports AND, OR and (t,n) threshold operations. The (t,n) threshold means that the secret information is divided into *n* parts, and at least *t* parts must be obtained to reconstruct the secret information. The AND operation can be regarded as an (n,n) threshold, and the OR operation can be regarded as an (1,n) threshold.

**Fig 3:**
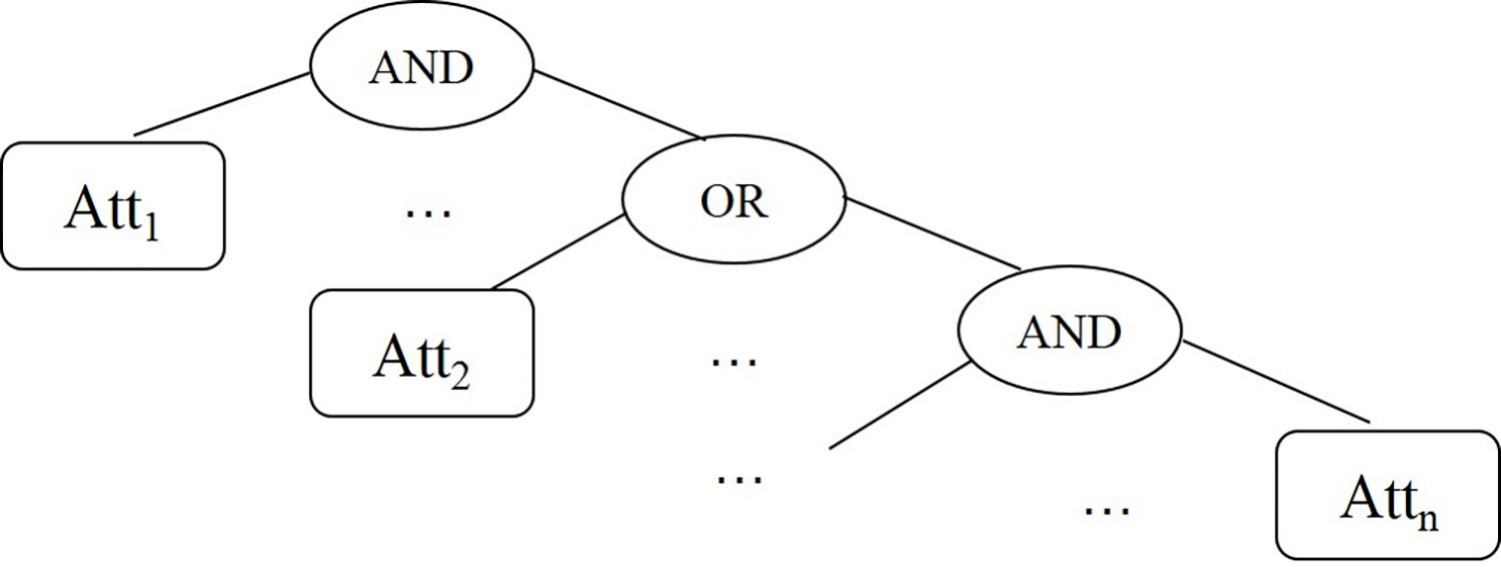
Access tree.

***Definition 3*** Access Tree. T is an access tree, each node is denoted by *x* in the tree, the number of child nodes of this node is denoted by n_*x*_, and its corresponding threshold value is denoted by k_*x*_. Each leaf node represents an attribute, and threshold values k_*x*_ = 1, n_*x*_ = 0. The relation between threshold value of non-leaf node and number of child node can be expressed by AND, OR and (t,n) threshold relation of attribute represented by leaf node, that is, k_*x*_ = n_*x*_ represents AND operation, k_*x*_ = 1 represents OR operation, 0<k_*x*_< n_*x*_ represents (t,n) threshold. Access tree is shown in Fig 3.

**Fig 4:**
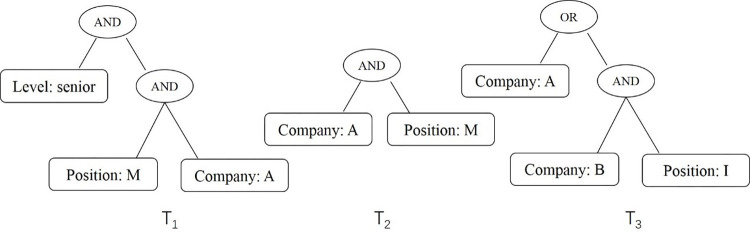
Example of access tree.

***Example 3***. In the above example, the identity files B_1_, B_2_ and B_3_ are constructed according to the privacy protection layer level, the access trees corresponding to the access policies are set as T_1_, T_2_ and T_3_ respectively, as shown in Fig 4.

For the identification file ciphertext B_1_, the access tree is T_1_, and the access user must meet three conditions at the same time, that is, he belongs to company A, his position is M and his level is senior. The user who meets the access structure can use the identification file B_1_ to remove dummy information. For example, Jim is an employee of Company A with position M and level senior, which meets the access structure. Tom is an employee of Company A with position M and level intermediate, which does not meet the access structure.

For the identification file ciphertext B_2_, the access tree is T_2_, and the access user must meet two conditions at the same time, that is, he belongs to company A and his position is M. The user who meets the access structure can use the identification file B_2_ to remove dummy information. For example, Jack is an employee of Company A with position M, which meets the access structure. Alice is an employee of Company A with position N, which does not meet the access structure.

For the identification file ciphertext B_3_, the access tree is T_3_, and the access user must meet the conditions, that is, he belongs to company A, or he belongs to company B and his position is I. The user who meets the access structure can use the identification file B_3_ to remove dummy information. For example, John is an employee of Company A, which meets the access structure. Martin is an employee of Company B with position I, which meets the access structure. Smith is an employee of Company B with position S, which does not meet the access structure.

### 3.6 Encrypt identification files and generate attribute keys

The data owner encrypts the identification file B_i_ to the ciphertext CB_i_ with the public key PK and the access structure tree T_i_. Then he sends the master key MK and access tree T_i_ to the trusted third party (TTP), while he sends the anonymous data set and identification file ciphertext to the data service provider (DSP). Data and attribute keys are stored separately in different servers to prevent privacy information from being leaked. When a privileged user u_i_ wants to use more accurate anonymous data, he sends his Certificate Attribute (CA) to TTP and requests the decryption key SK to identification file ciphertext. According to the access structure tree T_i_ and the attribute certificate CA_i_, the TTP generates a decryption private key SK_i_ of the identification file ciphertext CB_i_ and sends it to u_i_. Then u_i_ uses Sk_i_ to decrypt the identification file ciphertext CB_i_ to obtain the dummy information identification B_i_, and he can remove the dummy information from the anonymous data set to obtain relatively accurate data.

***Example 4***. In the above example, Jim is an employee of Company A, his position is M, and his level is high, his attributes meet the access structure tree T_1_, so he can obtain the decryption private key SK_1_ of the identification file ciphertext CB_1_. Tom is an employee of Company A, his position is M, and his level is intermediate, his attributes do not meet any access structure, then he cannot obtain the decryption private key.

Jack is an employee of Company A, his position is M, his attributes meet the access structure tree T_2_, so he can obtain the decryption private key SK_2_ of the identification file ciphertext CB_2_. Alice is an employee of Company A, her position is N, her attribute does not meet any access structure, then she cannot obtain the decryption private key.

John is an employee of Company A, Martin is an employee of Company B with position I, their attributes both meet the access structure tree T_3_, so they can obtain the decryption private key SK_3_ of the identification file ciphertext CB_3_. Smith is an employee of Company B with position S, and his attributes do not meet any access structure, then he cannot obtain the decryption private key.

### 3.7 User accesses data

After the data owner sends the anonymous data set M and the series identification file ciphertext CB_i_ to the data server, the user accesses the data through the data server. For an ordinary user, he can obtain anonymous data set M and identification file ciphertext CB_i_ from the data server, because there is no key to decrypt the identification file ciphertext, he can only use uniform data with high anonymity, it can better protect the privacy of the data owner.

**Fig 5:**
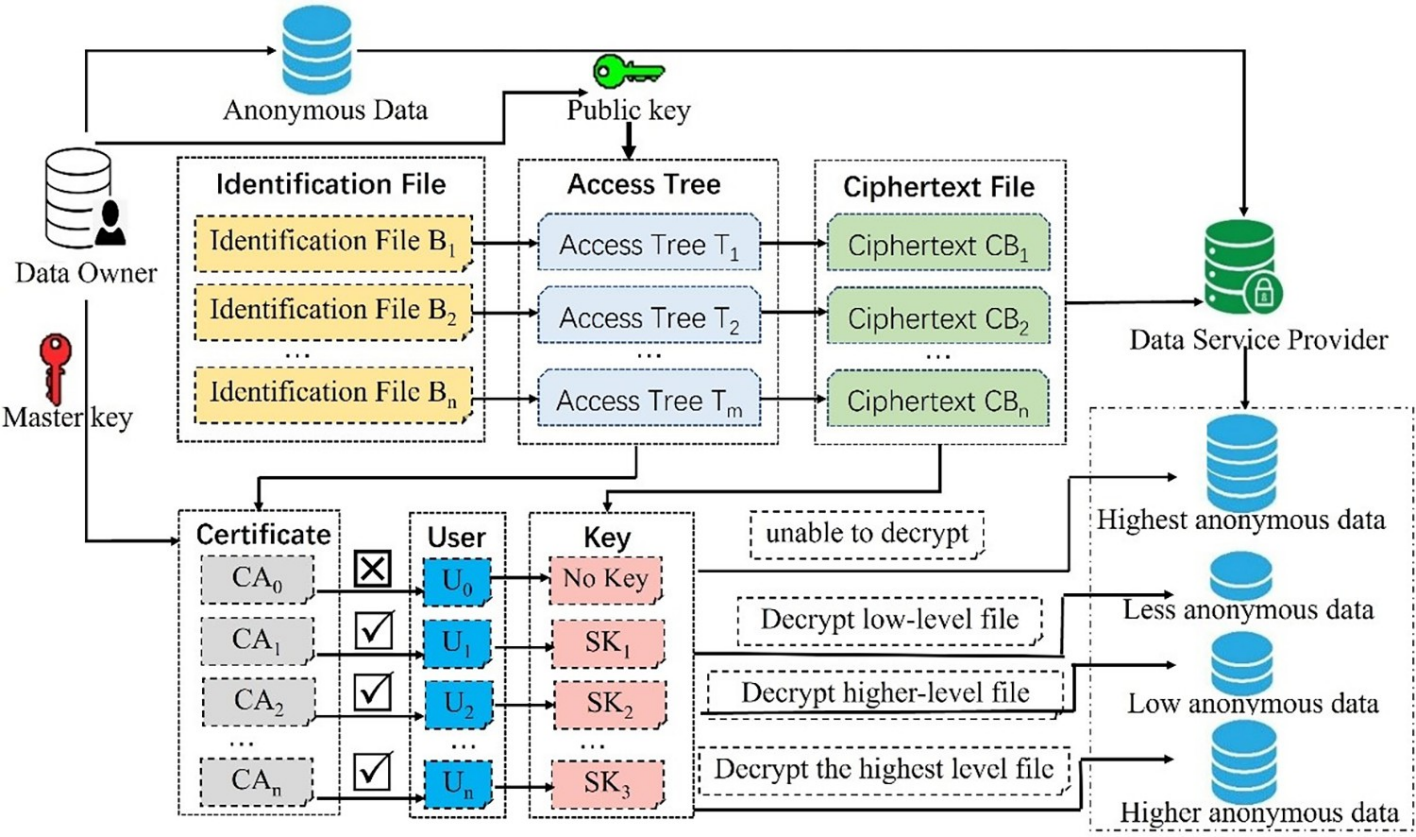
De-anonymization process.

For a privileged user, he can obtain the anonymous data set M and the identification file ciphertext CB_i_ from the data server, he can obtain the corresponding key SK_i_ from the trusted third party, he can decrypt the corresponding identification file ciphertext, he can perform de-anonymization operation and remove a certain amount of dummy information, he can obtain relatively accurate location data to improve the efficiency of data. The de-anonymization process is shown in Fig 5.

***Example 5***. In the above example, the attributes of ordinary users Tom, Alice and Smith are not satisfied with any access structure, they do not obtain any decryption private key of the identification file, so they cannot perform de-anonymization operation. They can only use the data set with high anonymity M = {s_7_, s_8_, s_9_, s_4_, s_5_, s_11_, s_2_, s_3_, s_10_}, so they cannot identify the real user location from M.

The attributes of privileged users John and Martin can satisfy the access structure T_3_, they can obtain the decryption private key SK_3_ of the identification file ciphertext CB_3_, and they can remove the dummy information {s_2_, s_3_, s_10_} from the anonymous data set M and obtain a more accurate data set M = {s_7_, s_8_, s_9_, s_4_, s_5_, s_11_}.

The attribute of privileged user Jack can satisfy the access structure T_2_, and he can obtain the decryption private key SK_2_ of the identification file ciphertext CB_2_, he can remove the dummy information {s_4_, s_5_, s_11_, s_2_, s_3_, s_10_} from the anonymous data set M, thereby obtaining a more accurate data set M = {s_7_, s_8_, s_9_}.

The attribute of privileged user Jim is satisfied with the access structure T_1_, and he can obtain the decryption private key SK_1_ of the identification file ciphertext CB_1_, then he can remove the dummy information {s_8_, s_9_, s_4_, s_5_, s_11_, s_2_, s_3_, s_10_} from the anonymous data set M, thereby obtaining a data set M = {s_7_} with only real users.

In the paper, we propose an access control strategy based on attribute encryption to encrypt identification files, and it forms the corresponding access structure according to the category of identification files. Only user whose attribute meets attribute conditions can decrypt identification files and de-anonymize data. When the data owner needs to adjust the user’s authority, it only needs to modify the access structure tree again, and then re-encrypt the identification file, which reduces the time cost for the data owner to regenerate and distribute the key. The specific execution process is shown in Algorithm 3.

Algorithm 3: Attribute Based Encryption and Decryption Algorithm

Input: Security parameter λ, Identification file B, Access tree T, User attribute S

Output: Identification file cipher CB, Decryption key SK, Decryption file CA

1 (MK, PK) = Gen(λ) *// Input security parameter λ to generate master key MK and public key PK*.*MK is kept by the data owner*, *PK is used to encrypt identification files*.

2 CB = Encrypt(PK,T,B) *// The identification file B is encrypted into ciphertext CB by public PK*, *accessing structure T*.

3 SK = KeyGen(MK,CA) *// The user’s private key SK is generated from the master key MK and user attribute value CA*.

4 B = Decrypt(CB,SK) *// Decrypt the ciphertext CB with the private key SK to obtain plaintext B*. *Decrypt() can only succeed if S satisfies T*.

The participants of attribute-based encryption access control system include data owner, trusted third party, user and service provider. The data owner owns the data and shares the data with other users through the service provider’s data service. The data owner is responsible for setting the access policy (access structure T), performing encryption algorithms to generate the identification file ciphertext bound to the policy, then he sends them to the service provider. The trusted third party is responsible for maintaining the correspondence between attributes and keys of each user, executing key generation algorithms to generate public keys PK and secret keys PK for data owners and generate attribute keys SK for access users. It is the only participant in the access control system that needs to be fully trusted by other participants. However, it is only responsible for sending the key and cannot access the ciphertext data. The user is the visitor of the data, if his attributes meet the policy requirements of the associated ciphertext, the trusted third party will generate the corresponding attribute key for him, then he can execute the decryption algorithm to obtain the plaintext of the identification file, and de-anonymize and precise access the anonymous data. The service provider is responsible for providing outsourcing storage of data and providing various operational services to users. It is honest and curious and will honestly perform various operations initiated by users, but it hopes to obtain more privacy content.

## 4. Safety analysis

The proposed method supports multi-level location privacy protection based on LBS, allowing users to share anonymous location data at various granularities. Initially, a mobile user submits their actual location to a reversible anonymity system managed by a trusted LBS provider. Such providers act as functional modules to facilitate both reversible processing and fine-grained location anonymity. In scenarios involving untrusted LBS providers, this reversible anonymity process can be implemented via a trusted third-party anonymizer.

The reversible de-anonymization process uses an attribute-based encryption access control policy. The data owner sets an access policy for each identifier file, generates a corresponding attribute access structure tree, and encrypts the identifier files using this tree as a parameter to generate ciphertexts. Users whose attributes align with the structure tree requirements can request a decryption key from the trusted third party to perform de-anonymization operations and access more precise data at specified privacy protection levels. The security services implemented by the proposed method cover five main areas.

### 4.1 Confidentiality

A user’s real location is blended with randomly added dummy locations to ensure the real location remains private. Multi-level location privacy protection is achieved after adding varying amounts of dummy information at different hierarchical levels *N*. Data users of varying trust levels receive data with corresponding levels of anonymity but are unable to access the real location information. Ordinary users cannot decrypt encrypted identifier files to remove dummy information; instead, they access uniformly anonymized data. This facilitates robust, multi-level, multi-granularity protection of private location data. Therefore, the proposed method can ensure that the user’s privacy information is not leaked.

### 4.2 Multilevel privacy preserving

According to the privacy protection requirements, multi-level location privacy protection is achieved after adding varying amounts of dummy information at different hierarchical levels N. The lower level of anonymity, the less dummy information is added. The higher level of anonymity, the more dummy information is added. The usage, encryption and decryption of dummy information identifier files are managed through an attribute-based encryption access control system. Only privileged users who align with the access structure tree criteria can obtain decryption keys, ensuring the privacy of both the keys and the real location data. Data users of varying trust levels receive data with corresponding levels of anonymity, it achieves multi-level location privacy protection.

### 4.3 Restorability

In the paper, all dummy information which is added to anonymous set by the proposed method is marked hierarchically in identification file. For privileged users whose attributes align with the structure tree requirements can request a decryption key from the trusted third party to perform de-anonymization operations, they can remove dummy information in a certain extent, recover relatively accurate location data, and use data efficiently, which achieves the restorability. of location data.

### 4.4 Authentication

The proposed method can achieve the identity authentication of privileged users through access control mechanism based on attribute encryption. The data owner sets the access control policy and generates the access structure tree. The trusted third party verifies the attribute certificate of the privileged user to confirm the user’s identity. When his attribute meets the access structure conditions, he can obtain the decryption key and perform de-anonymity processing. Trusted third party can prevent illegal users from obtaining privacy information by verifying user’s attribute certificate.

### 4.5 Availability

The proposed method can ensure the availability of data, on the one hand, in the process of data anonymity, by constructing undirected graph and adjacency table, using hash function to select adjacent position points, adding different dummy information in different levels, which makes anonymous data to be highly available. On the other hand, privileged user whose attribute is satisfied with the access structure can de-anonymize anonymous data set to improve the accuracy of data use and ensure the higher data availability.

In conclusion, the proposed method can guarantee the confidentiality, availability and authentication of data security services, it can not only provide multi-level privacy protection, but also achieve the recovery operation of location data after anonymity.

## 5. Experimental evaluation

In this part, we evaluate the performance of proposed method mainly from the anonymity success rate, data efficiency, anonymization computational overhead, de-anonymization computational overhead and the entropy of location set. Compared with other methods to verify the feasibility and effectiveness.

### 5.1 Data and experimental setup

#### 5.1.1 Experimental data

The experimental data used is Geolife dataset [24–26], which includes GPS trajectory data of 182 users over five years. Each location point contains latitude, longitude, time and other information, including 24,876,978 location points, 18,670 trajectories, with a total duration of 11,129 days and a total distance of 1,292,951 kilometers. The most majority of these data sets come from Beijing, with little data coming from Europe or the United States. The data includes a variety of social activities, such as work, home, entertainment and sports activities.

#### 5.1.2 Experimental setup

The experimental environment is Intel(R) Core(TM) i7 CPU@3.60 GHz, 32GB memory, Windows 10–64 bit operating system, and the specific method is implemented by Python 3.7. Privacy protection level i_N_ = 10, i = 1,2,…,10, i.e. the privacy protection level is set to 10, privacy protection parameters *k*_i_ and spatial tolerance rate *d* are set to:

$$k_{i}=10N,\;d=\lceil {40k}_{i}\sqrt{N}\rceil ,\;N=1,2\ldots 10$$

The spatial tolerance *d* is a function of the privacy protection parameter *k*_i_ and the privacy protection level *N*, where *d* is in meters (m) and the time tolerance *t* is fixed at 20 seconds. All experiments were repeated 100 times and the average was considered the results.

### 5.2 Experimental results and analysis

The proposed method (referred to from here on as the BME-LPP was compared against the method proposed in paper [9] (referred to from here on as the RPLE) and the method proposed in paper [27] (referred to from here on as the TPS). The RPLE method uses the spatiotemporal anonymity model to reversibly disturb user location information to achieve reversible location privacy protection for mobile users. The TPS method combines k-1 similar trajectories with real trajectories to form a false trajectory region to realize k-anonymity of a given location.

#### 5.2.1 Anonymity success rate

**Fig 6:**
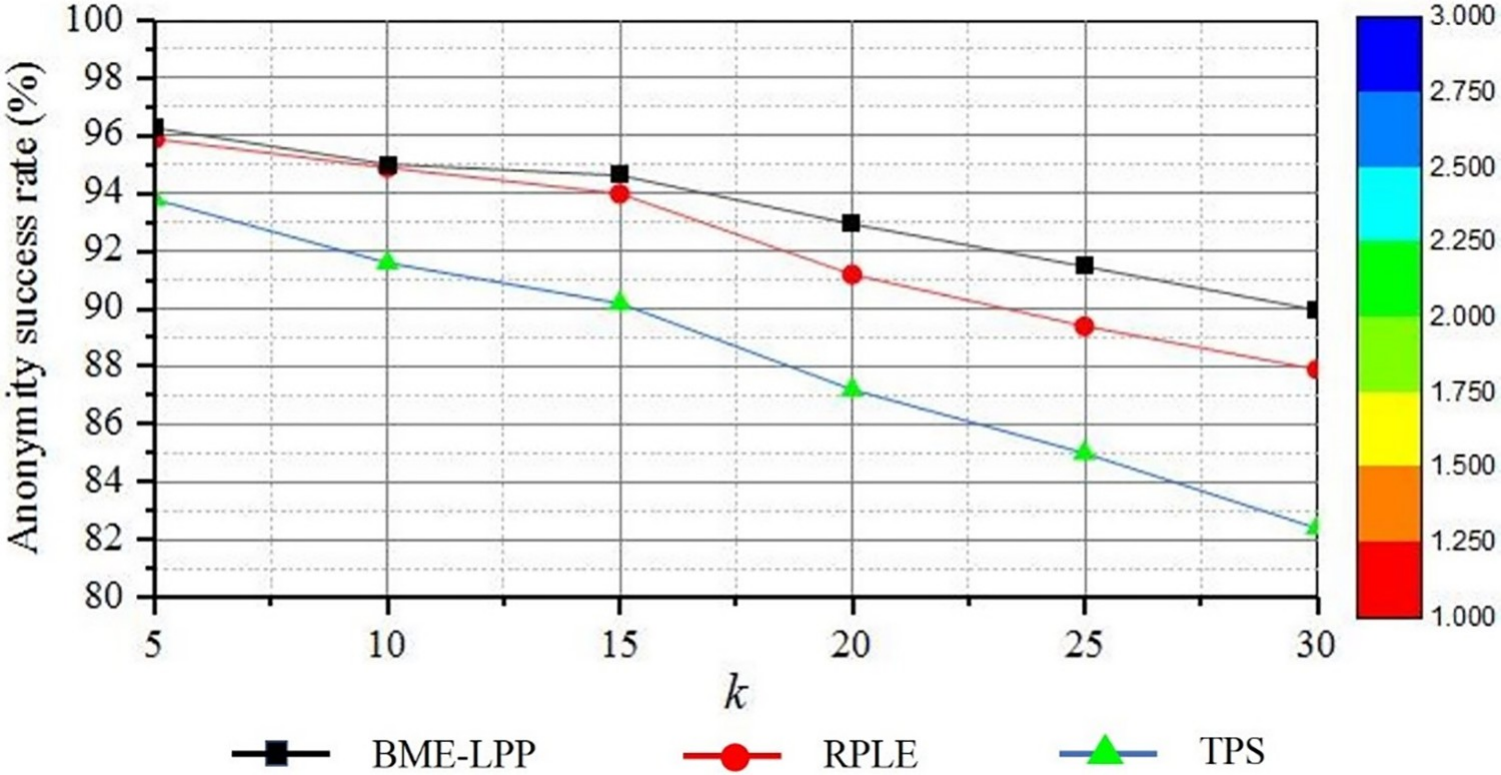
Comparison on anonymization success rate.

Anonymity success rate is used to measure the ability of privacy protection methods to resist attacks. If the anonymity success rate is high, it is difficult for attackers to anonymously identify the true location of users. Fig 6 shows a comparison of anonymization success rates for the three methods. As can be seen from the figure, the anonymity success rate is decreased with the increasing of *k*. Because with the increase of *k*, more and more dummy information needs to be added in anonymous sets. Within the specified spatial tolerance *d*, it is increasingly difficult to select the enough qualified locations, so the anonymity success rate will be reduced.

However, it can be seen from the figure that the proposed BME-LPP method achieves the highest anonymity success rate. On the one hand, the proposed method ensures that the user’s real location information is not leaked by adding position-related dummy information hierarchically, dummy information selection is still carried out by adding adjacent segments, security is also ensured by asymmetric anonymous encryption is to achieve security. On the other hand, by asymmetric encryption and attribute-based access control to encrypt the de-anonymized identification file before transmission, it can ensure that the actual location information of the user is not leaked. Therefore, the anonymous success rate of the proposed method is higher than other methods. The RPLE method proposes a method to construct anonymous sets by selecting the position points associated with the current anonymous set as dummy position points, which can also provide high anonymity success rate. However, its de-anonymization process is not encrypted, which will lead to the disclosure of private information. TPS method divides the real trajectory into sub-trajectories according to time sequence, then it searches for similar trajectory segments in historical trajectory data set, and it assembles similar sub-trajectory segments into false trajectory of user, it cannot resist similarity attacks, so its privacy protection effect is relatively low.

#### 5.2.2 Data efficiency

Data utilization efficiency mainly refers to the utilization efficiency of published location data after anonymity is used by the third party, and it also reflects the loss rate of location information in the process of anonymity. In the paper, data efficiency is indirectly measured by calculating the information loss of published location data, which is measured by calculating the ratio of the size of the final anonymous region to the size of the maximum allowable spatial region. The greater loss of information from the published data set, the less efficient data will be used. For privileged users, the proposed method can perform de-anonymization operation according to user’s permission, and it can partially or completely remove dummy information from data set, so the data availability can achieve 100%. For ordinary users, because it is impossible to decrypt the ciphertext of the identification file, it is impossible to remove dummy information and achieve complete data utilization. Therefore, in this section, we will discuss data availability of ordinary users and privileged users separately.

**Fig 7:**
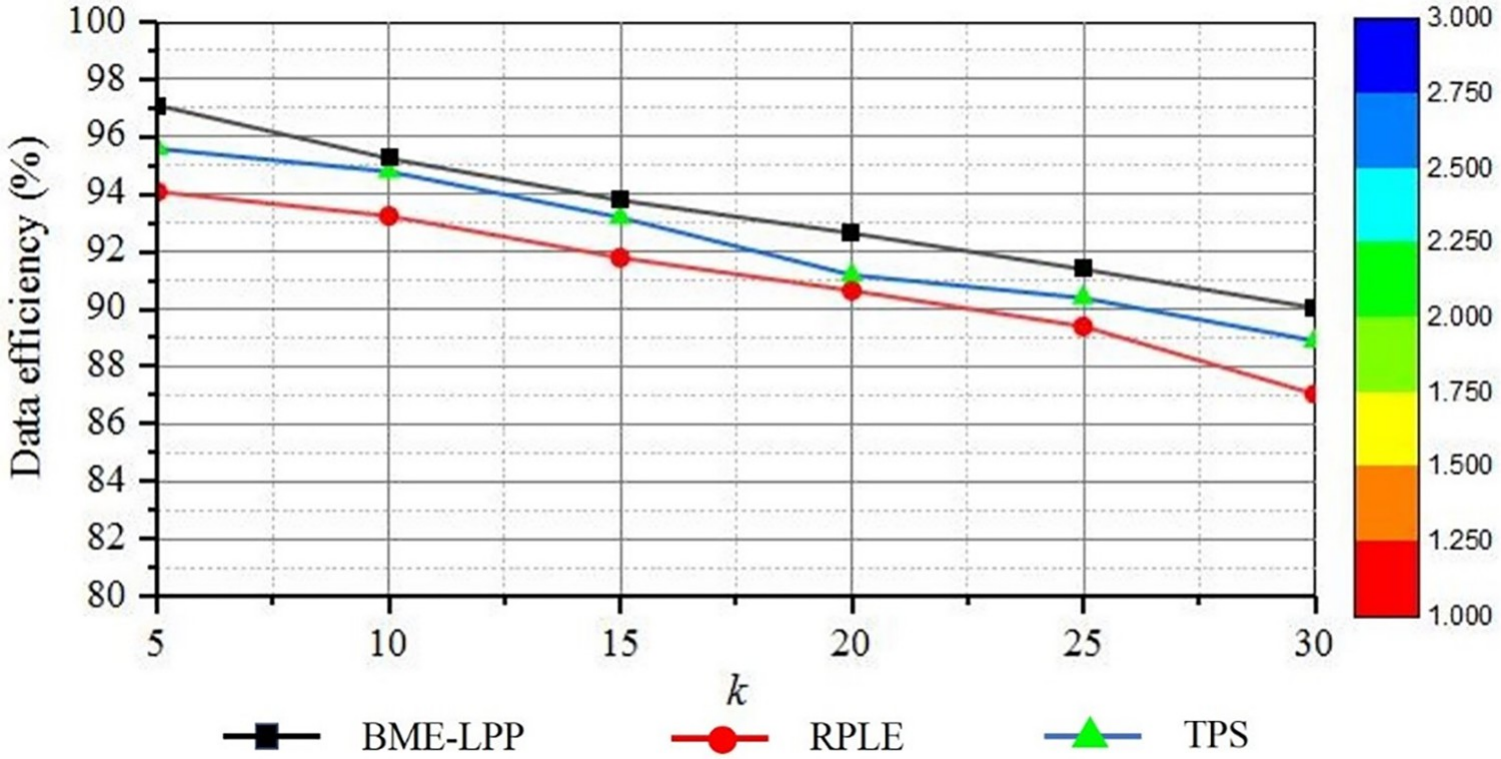
Anonymized data efficiency.

Fig 7 shows that the anonymized data utilization efficiency is decreased as the privacy preserving parameter *k* is increased. With the increase of privacy protection parameters, the more dummy information is added. Then the disturbance to the data set will be increased, which will reduce the utilization of the data set. As can be seen from the figure, the proposed method BME-LPP achieves the highest data utilization. BME-LPP method constructs dummy data set by constructing position adjacency table to select location points associated with original location points, so that data characteristics in data set are consistent, and it can achieve high data availability. The RPLE method determines the selected position by generating a series of pseudo-random numbers with a key, and it uses a local expansion algorithm to achieve perturbation of the true position, the anonymous set formed has greater diversity. TPS method uses Euclidean distance to calculate the linear distance between positions, and there is some error in distinguishing similar positions. Therefore, their data availability is relatively lower.

**Fig 8:**
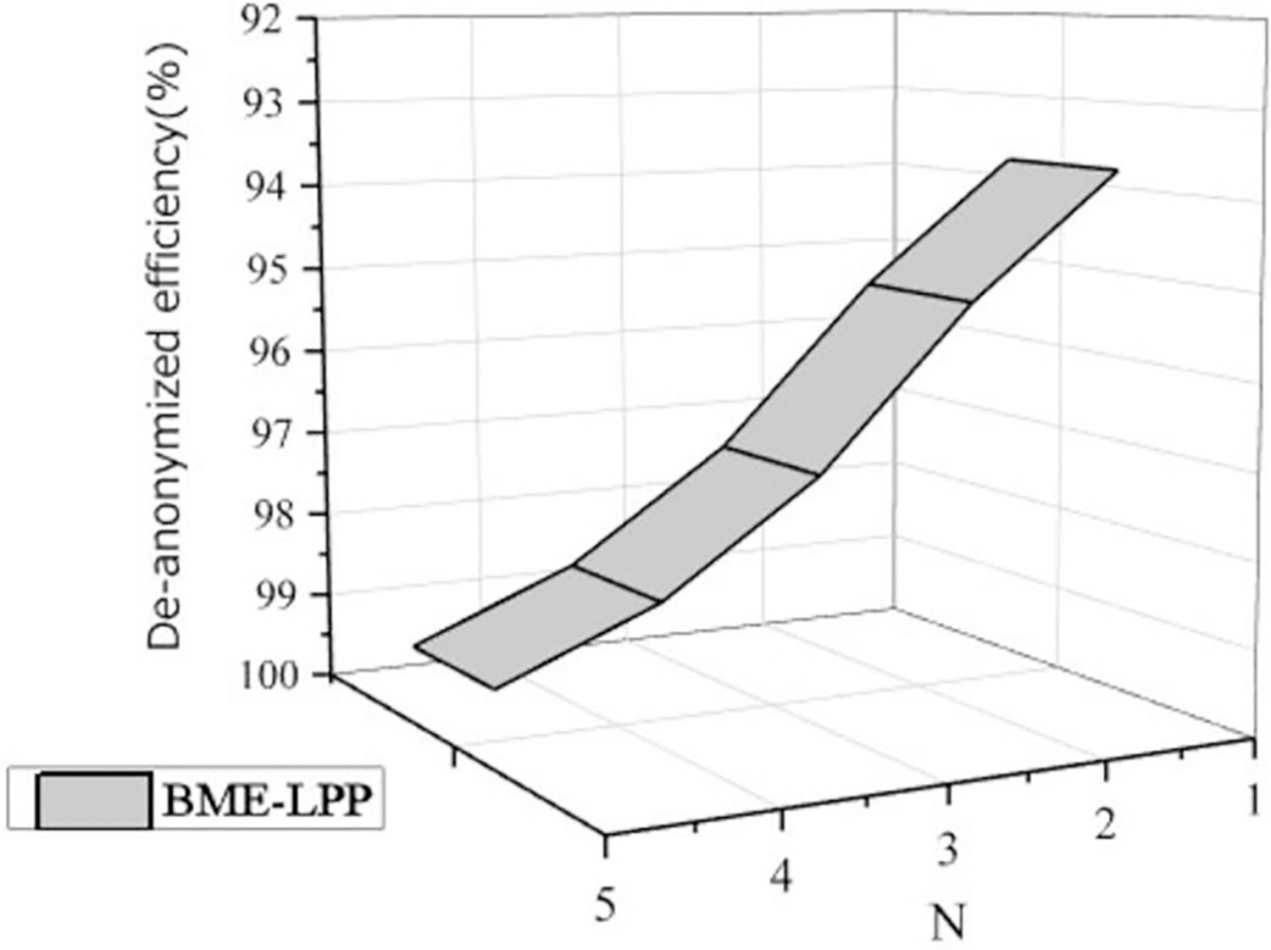
De-anonymized data efficiency.

For privileged users, BME-LPP method can eliminate dummy information and achieve higher data availability. Fig 8 shows that the de-anonymized data utilization efficiency is increased as the de-anonymization level is increased. In the experiment, the de-anonymization level is set to N = 5, the initial privacy protection parameter is set to k_1_ = 10, and the privacy protection parameter increment level is 10 for each anonymity level, i.e. *k*_i_ = 10+(i-1)10 = 10•i,(1≤i≤N). As can be seen from the figure, when the de-anonymization level is 1, the dummy information removed is relatively small, and the data utilization efficiency is relatively low. When the de-anonymization level is increased, the more disinformation is removed, the more data utilization achieved. When the de-anonymization level is 5, the highest data utilization is achieved.

#### 5.2.3 Anonymization computational overhead

Anonymization computational is an important factor of service quality for user, and it is the most intuitive factor to measure the effect of experiments. Therefore, under the condition of anonymity, the smaller computational overhead, the better privacy protection.

**Fig 9:**
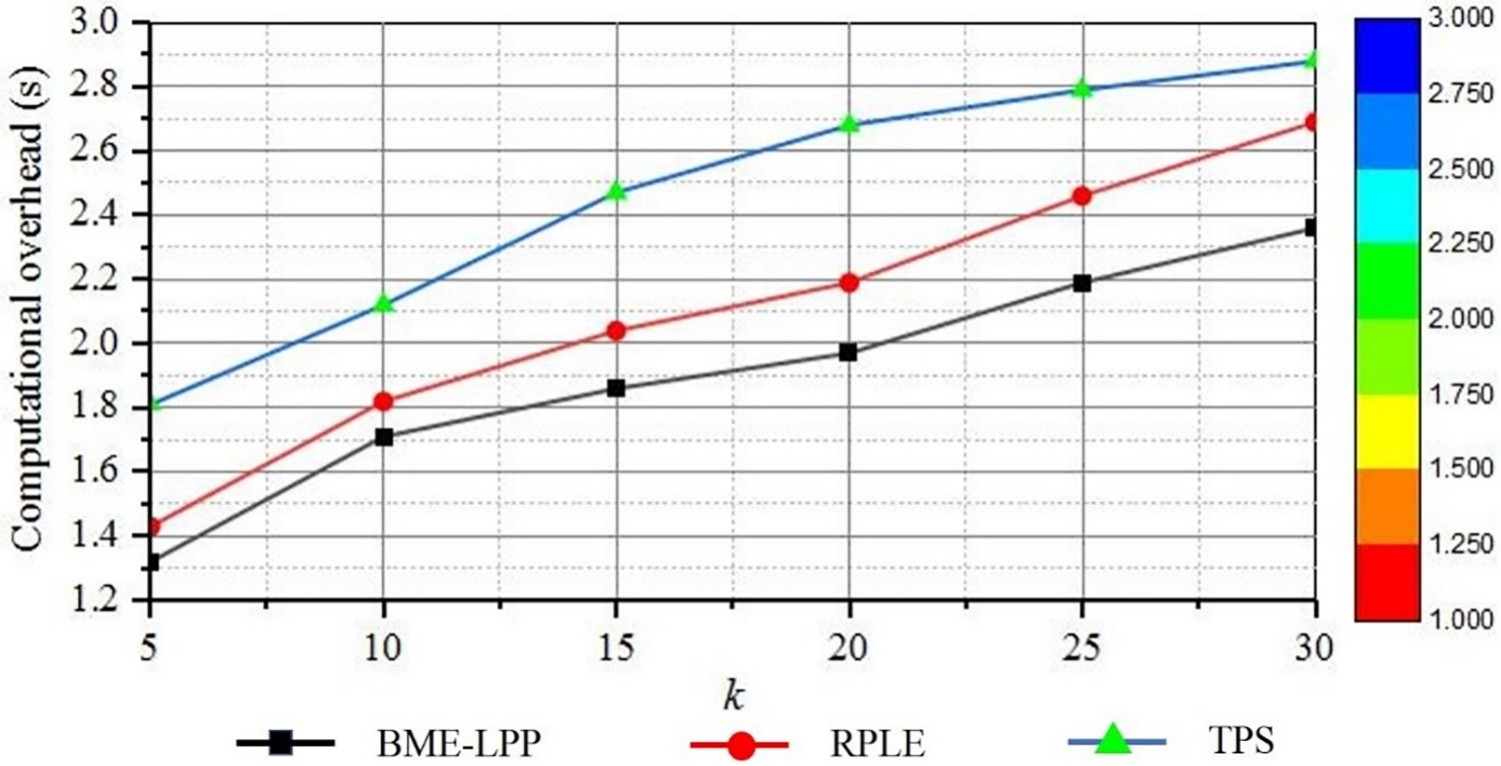
Anonymization computational overhead.

Under the condition of anonymity, the smaller computational overhead, the better privacy protection. Fig 9 shows a comparison of the computational overhead of the various methods. It can be seen from the figure that the computational overhead of all privacy preserving methods is increased with the increase of privacy preserving parameter *k*. Because with the increasing of *k*, more and more dummy information needs to be added in the anonymous set, and more and more qualified positions need to be selected, so the computational overhead will be increased continuously. It can also be seen from the figure that the computational overhead of the proposed BME-LPP method for anonymous processing is relatively low. Because the undirected graph and adjacency table are constructed to select dummy information, it can reduce the computational overhead, and the encryption of de-anonymized identification files can be performed offline, which also makes the computational overhead is relatively small. However, the RPLE needs longer anonymization runtime to construct collision-free links instantly, and it also needs to generate correlation keys in the anonymization process to ensure the reversibility of deanonymization processing, so the computational overhead is higher than the proposed BME-LPP method. TPS method needs to calculate semantic similarity, spatial similarity and temporal similarity between position trajectories, and its time complexity is the highest.

**Fig 10:**
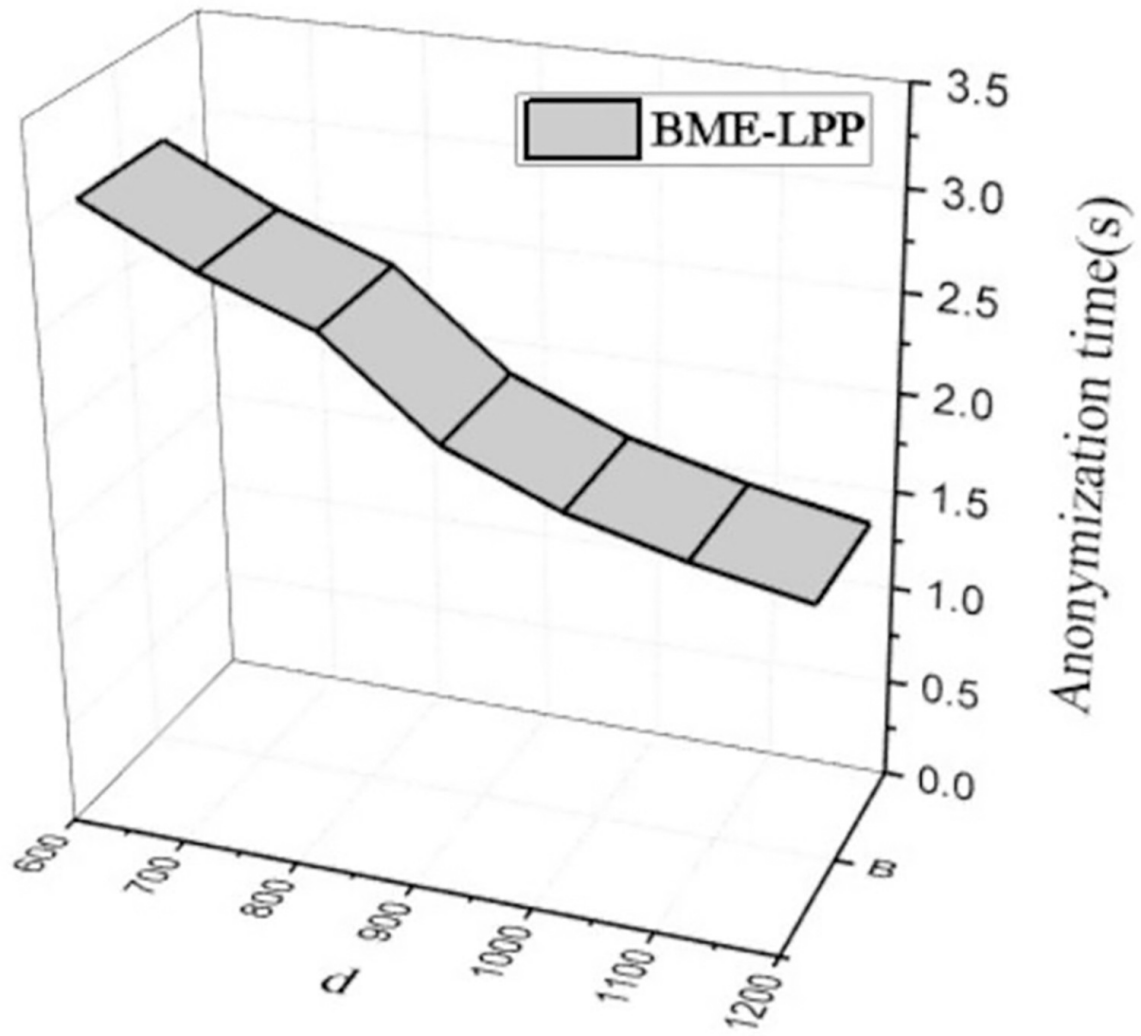
Anonymization computational overhead.

Fig 10 shows that the computational overhead of anonymization is decreased with increasing of spatial tolerance *d*. With the increasing of *d*, the range of dummy locations is further expanded, and it is easier to select locations that meet the condition to construct anonymous sets, so the computational overhead is decreased. However, when the privacy parameter *k* is a constant, the computational overhead is not decreased significantly with the increasing of *d*, because the expansion of spatial region will increase the range of selected dummy location information in some extent, but it will not make the anonymity condition easier. As can be seen from the figure, there is not much difference in system computational overhead when *d* = 1000 and *d* = 1200. Therefore, the computational overhead of the proposed BME-LPP method is decreased continuously with the increase of *d*, but it ss not decreased indefinitely and converged gradually to a certain critical value.

#### 5.2.4 De-anonymization computational overhead

**Fig 11:**
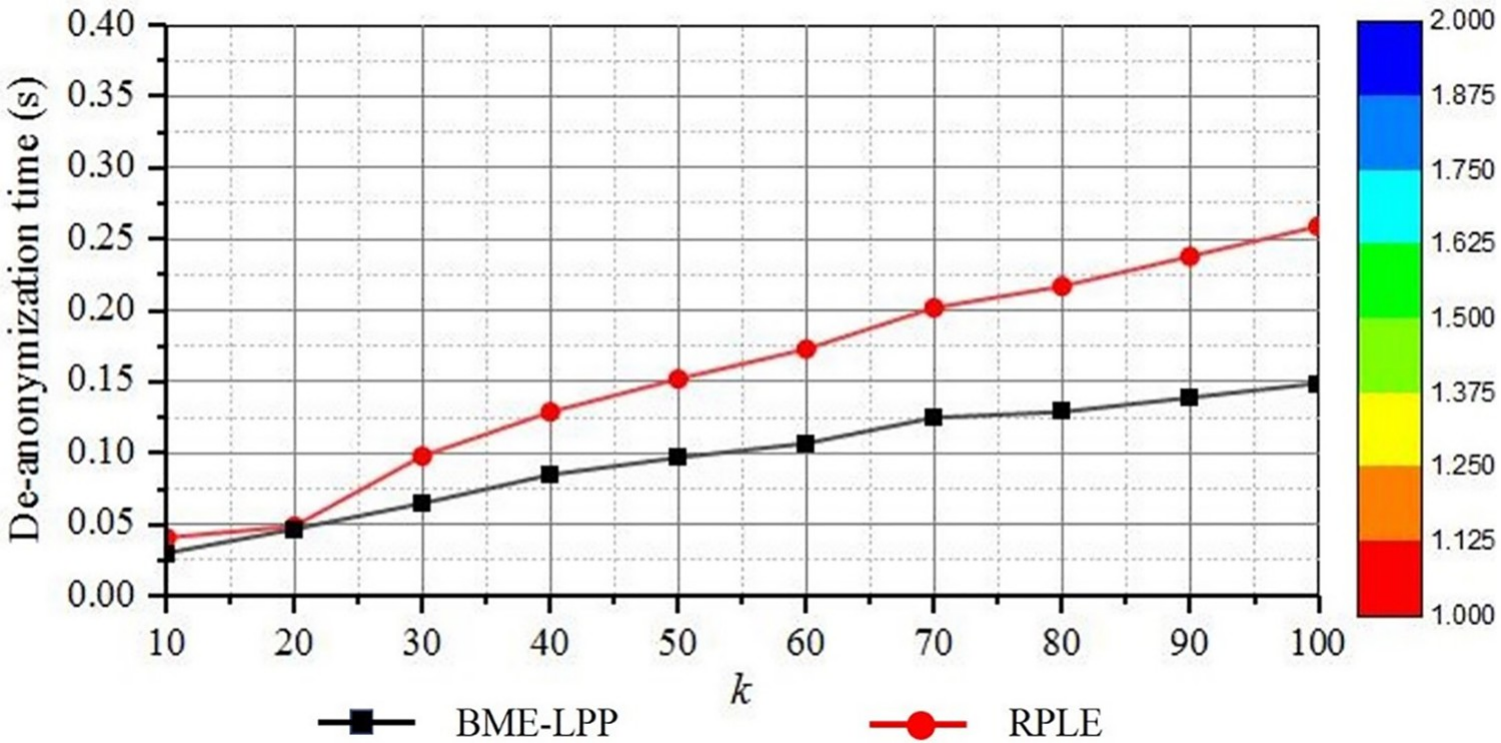
De-anonymization computation overhead.

De-anonymization computational overhead is also an important factor to measure the quality of service. Under the condition of ensuring privacy protection effect, the smaller computational overhead, the higher efficiency of privacy protection method is. Fig 11 shows that the computational overhead of de-anonymization is increased slowly when the anonymity parameter *k* is increased.

When k is increased, more dummy information is added to the anonymous set. Then in the deanonymization phase, more dummy information will be removed, which will lead to increased computational overhead.

It can also be seen from the figure that the de-anonymization efficiency of the proposed method BME-LPP is better than the RPLE method. Because BME-LPP can directly remove dummy information according to dummy information identification files, there is no complicated calculation in the whole de-anonymization process, so its execution effect is less. Although the computational overhead is increased slightly, the gap of them is extremely small for the overall system. Because in the de-anonymization process, the marked dummy information in the deanonymized identification file is directly removed, the computational overhead is inherently small in the process, and the difference of them can be ignored. However, RPLE method needs to use the secret key to calculate and select the dummy position information through the transformation matrix, which will increase the time complexity.

**Fig 12:**
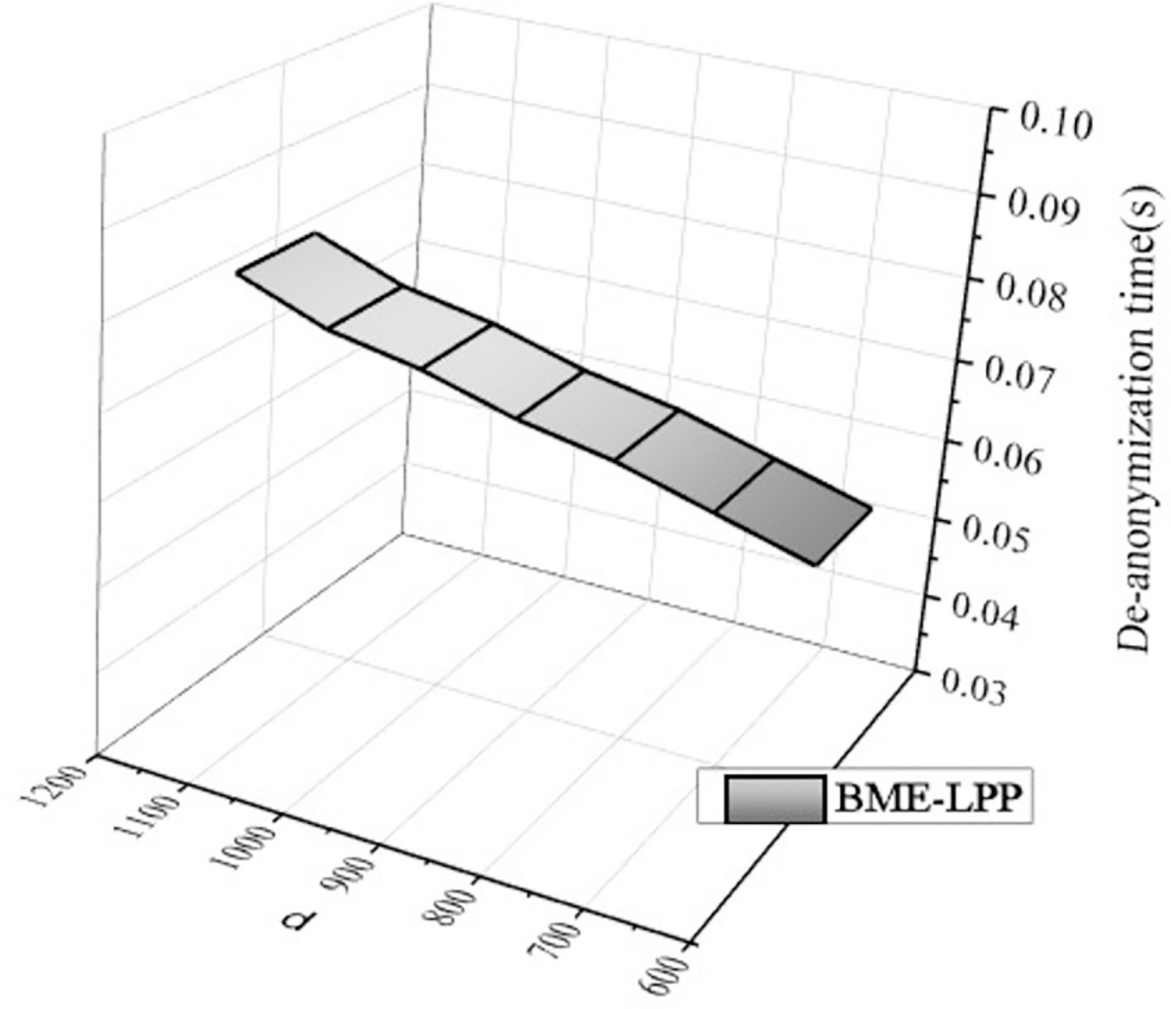
De-anonymization computation overhead.

Fig 12 shows that the computational overhead of de-anonymization is increased slightly as the spatial tolerance *d* is increased, but it is not significantly. When the anonymity parameter *k* is a constant, the de-anonymization time does not change with *d*. When the *d* is increased, the range of dummy location selection is expanded, it is easier to select locations which meet the condition to construct anonymous sets, but it has little effect on de-anonymization processing, and the computational overhead is mainly used to remove the dummy location information. Therefore, the computational overhead of the proposed BME-LPP method does not change much with the increasing of spatial tolerance *d*.

#### 5.2.5 Location set entropy

Location set entropy is used to measure the uncertainty of user being identified in anonymous set. The higher entropy, the higher similarity of user locations. The greater uncertainty of the user’s true information is inferred by the attacker, the better effect of privacy protection. The formula for calculating the entropy of location set is: $H=-\sum_{i=1}^{k}p_{i}∙lnp_{i}$.

**Fig 13:**
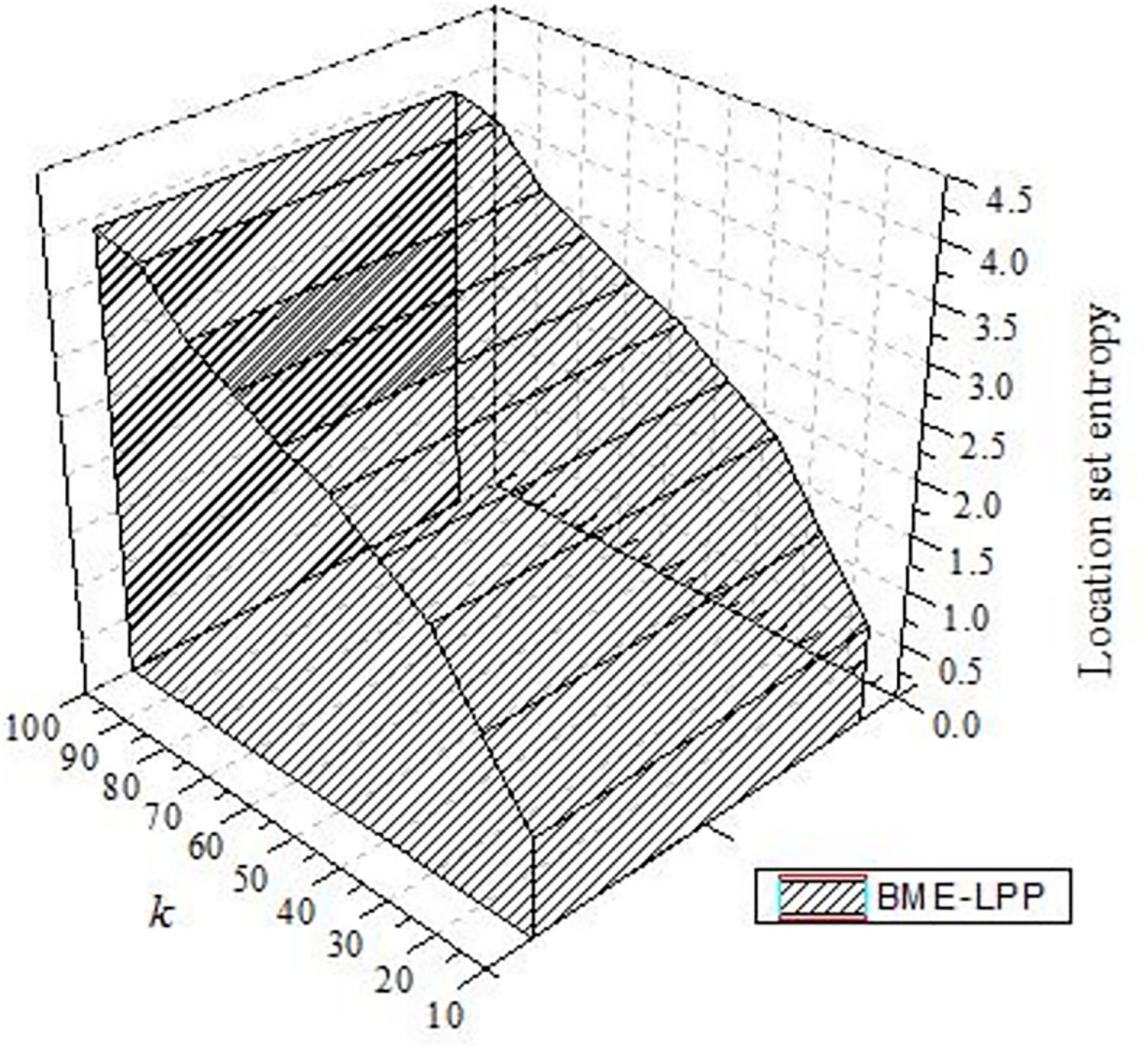
Location set entropy.

Fig 13 shows that the entropy of location set of proposed method is increased with the increase of the privacy preserving parameter *k*. The higher the entropy, the less probability an attacker would be able to identify the user’s true location. Because the higher similarity of location points in the set, the greater uncertainty that true location is identified, the better privacy protection effect.

### 5.3 Experimental results

Experimental results show that the proposed BME-LPP method can provide bidirectional reversible and multi-layered privacy protection. It can solve the encryption management of de-anonymized identification files and the generation and distribution of attribute keys by using access control method based on attribute encryption. The BME-LPP method can refine anonymous data in different degrees by using the dummy information identification files, and to recover more accurate user data, so it can provide the higher data utilization while protecting user location privacy. At the same time, although the proposed method can provide multi-level privacy protection, it only publishes an anonymous data set, and only identifies dummy information hierarchically in the anonymization process, so the processing time will not be increased. Moreover, the encryption transmission of identification file can be carried out offline or independently, so the computational overhead is smaller than the encryption methods in reference [2, 9].

Although reference [2] provides good privacy protection effect, the query content is encrypted and decrypted, which will increase the response time to a certain extent. Reference [5] proposes an L-clustering algorithm based on differential privacy protection, which clusters user’s long-time stay points, high-frequency stay points and sensitive location points, but the data utilization rate is lower. Reference [9] can provide multi-level privacy protection, it creates a list of transformations for each location point, which is spatially complex. Dynamically maintain the adjacent position relationship of each location point, time complexity is high. Reference [2] proposes a differential privacy protection method, it adds noise to the resident points and centroids in the cluster, which can provide a certain degree of privacy protection, but the addition of noise reduces data utilization. Reference [18] selects pseudo-offset locations to construct secure anonymous sets, reference [20] improves privacy protection by replacing sensitive staying areas of users, but their computational overhead is large. A comparison of the proposed BME-LPP method with other location privacy preserving methods is shown in Table 2.

**Table 2:** Comparison with related method.

Ref.	Year	Privacy preserving method	Multilevel preserving	Data restorability	Privacy preserving and data utility	Execution efficiency
BME-LPP	/	Add and remove dummy location hierarchically	Yes	Yes	Good privacy preserving and high data utility	High
[2]	2023	Encryption and Substitution of sensitive semantic locations	No	No	Better privacy preserving, but low data utility	Low
[5]	2022	Sensitive Location L-Clustering and add noise	No	No	Good privacy preserving, but low data utility	Medium
[9]	2017	Add and remove dummy location through transformation matrix	Yes	Yes	Good privacy preserving and data utility	Medium
[2]	2023	Differential Privacy	No	No	Better privacy preserving, but low data utility	Low
[18]	2022	Use dummy locations to construct anonymous sets	No	No	Good privacy preserving, but low data utility	Low
[20]	2022	Replacement of user’s sensitive stay area.	No	No	Good privacy preserving, but low data utility	Low

## 6. Conclusion and next work

In the paper, in order to solve the problem that the single-layer, one-way and coarse-grained privacy protection for location-sensitive data cannot meet the actual privacy protection requirements of users, a bidirectional multi-layered location privacy protection method based on attribute encryption is proposed. It can settle the coarse-grained privacy protection problem caused by the "all" or "none" rigid privacy protection. The proposed method can achieve bidirectional processing of location privacy protection, including both anonymized privacy protection and de-anonymized data availability refinement. When dummy information is added to protect user data anonymously, a series of identification files marked with dummy information are generated, and privileged users can use them to carry out multi-level de-anonymization processing and obtain more accurate user data. The proposed method uses Hash function to generate random numbers and select dummy information to improve privacy protection effect. It uses attribute-based encryption access control method to encrypt and manage dummy information identification files. It generates decryption keys based on user attributes, so that users with different trust levels can perform different de-anonymization operations. It can improve the efficiency of information processing while providing multi-level privacy protection. Experimental results on real data sets show that proposed method has low computational overhead, high anonymity success rate and data utilization efficiency.

However, the propose method is mainly applicable to the scenario where there is only one trusted third party in the system, while there may be multiple trusted authority in distributed scenarios, multiple attribute authorities need to be supported simultaneously, and each authority can issue attribute keys independently to support distributed multi-attribute application scenarios. Therefore, our next work is to research reversible multi-layer encryption schemes for distributed multi-attribute application scenarios.
